# Prevalence and correlates of facemask usage during the second wave of COVID-19 pandemic in Uganda

**DOI:** 10.1371/journal.pgph.0002569

**Published:** 2025-02-07

**Authors:** Nelson Onira Alema, Christopher Okot, Emmanuel Olal, Eric Nzirakaindi Ikoona, Freddy Wathum Drinkwater Oyat, Steven Baguma, Denish Omoya Ochula, Patrick Odong Olwedo, Johnson Nyeko Oloya, Francis Pebalo Pebolo, Pamela Okot Atim, Godfrey Smart Okot, Ritah Nantale, Judith Aloyo, David Lagoro Kitara

**Affiliations:** 1 Gulu University, Faculty of Medicine, Department of Anatomy, Gulu City, Uganda; 2 Gulu Regional Referral Hospital, Gulu City, Uganda; 3 Uganda Medical Association (UMA), UMA-Acholi branch, Gulu City, Uganda; 4 Yotkom Medical Centre, Kitgum, Gulu City, Uganda; 5 ICAP at Columbia University, Freetown, Sierra Leone; 6 District Health Office, Lamwo local government, Lamwo, Gulu City, Uganda; 7 Hospital Director, Yumbe Regional Referral Hospital, Yumbe, Arua, Uganda; 8 Moroto Regional Referral Hospital, Moroto, Mbale, Uganda; 9 Gulu University, Faculty of Medicine, Department of Reproductive Health, Gulu City, Uganda; 10 St. Joseph’s Hospital, Kitgum, Gulu City, Uganda; 11 Dr. Ambrosoli Memorial Hospital, Kalongo, Agago, Gulu City, Uganda; 12 Busitema University, Faculty of Health Sciences, Department of Maternal and Child Health, Mbale, Uganda; 13 Rhites-N, Gulu City, Uganda; 14 Gulu Centre for Advanced Medical Diagnostics, Research, Trainings, and Innovations (GRUDI BIONTECH INITIATIVE), Gulu City, Uganda; 15 Gulu University, Faculty of Medicine, Department of Surgery, Gulu City, Uganda; PLOS: Public Library of Science, UNITED STATES OF AMERICA

## Abstract

The World Health Organization (WHO) and the United States Centers for Disease Control and Prevention (US CDC) documented wearing facemasks in public as one of the most important prevention measures to limit COVID-19 spread. Considering this, WHO and the US CDC developed guidelines for wearing facemasks in public. This study aimed to determine the prevalence and correlates of facemask wearing during the COVID-19 pandemic in northern Uganda. We conducted a cross-sectional study on 587 adults across nine districts in northern Uganda, across 24 high-volume health facilities offering free COVID-9 vaccines. Respondents were selected from the health facilities using a single-stage systematic sampling method. Data was collected in a face-to-face questionnaire interview with an internal validity of Cronbach’s α = 0.72 and entered into Excel. A local Institutional Research Board (IRB) approved the study, and Stata 18 was used for data analysis using Modified Poisson Regression to generate prevalence ratios (PR) and adjusted prevalence ratios (aPR), with a p-value set at < 0.05. The reported prevalence of facemask wearing in public among respondents was high [88.7%,95%CI:86%,-91%]. A multivariate analysis found that obese respondents and those who were receptive (agreed) to the lockdown measures were respectively,1.12 times more likely to wear facemasks [aPR = 1.12,95%CI:1.04–1.19;p < 0.01], and1.23 times more likely to wear facemasks [aPR = 1.23, 95%CI:1.07–1.41;p < 0.01]. The most significant finding from this study was the high prevalence of self-reported facemask wearing among adult community members in northern Uganda. The correlates of wearing facemasks were, being obese and agreeing with the presidential directives on the lockdown measures. Although this prevalence is within acceptable rates, the strict enforcement of the practice by security forces has raised concerns among many community members and human rights advocates. We recommend more studies on communities’ perspectives on the challenges and benefits of facemask-wearing after the COVID-19 pandemic.

## Introduction

On March 11, 2020, the World Health Organization (WHO) declared an outbreak of a new and fast-spreading respiratory disease, COVID-19 [1] which was caused by severe-acute-respiratory-syndrome-coronavirus-2 (SARS-CoV-2) [2]. As of March 2023, there were 761,402,282 confirmed cases of COVID-19 worldwide; 9,514,948 in Africa, with Uganda contributing 170,525 cases (data from WHO COVID-19 dashboard as of 29^th^ March 2023) [3]. Much as Africa had fewer cases of COVID-19, the WHO warned of consequences likely to result from community transmissions in low-income countries with weak health systems [3]. Indeed, community transmissions of COVID-19 occurred in the African continent with severe consequences [4].

On June 6, 2020, the Director General of WHO recommended and confirmed the use of facemasks as a preventive measure for COVID-19 [5]. In addition, the use of non-pharmaceutical measures for pandemic influenza in non-healthcare settings, for example, social distancing measures has been reported and used during many epidemic controls including COVID-19 [6].

Coronavirus disease 2019 (COVID-19) is a worrying respiratory tract disease resulting from infection with SARS-CoV-2 [7]. As the WHO recommended, several pandemic response activities were instituted across the globe to curb the spread of coronavirus (COVID-19) [8]. Social distancing (SD)/Physical distancing (PD), hand hygiene, mouth covering when coughing and sneezing, avoidance of touching the face, and wearing facemasks were critical non-pharmaceutical public health interventions that proved effective in reducing the spread of SARS-CoV-2 [8–10].

Facemask wearing in public situations was documented by the WHO and CDC as one of the most important prevention measures that limited the acquisition and spread of COVID-19 [11,12]. In light of this, the WHO and CDC developed guidelines for using facemasks in public settings [11,12].

Published studies have shown that wearing facemasks to control the spread of infectious diseases had several advantages, including simple operation, strong sustainability, high health benefits, and good health economic benefits [10,13–15]. In addition, other studies showed that facemasks used by the general public were of potentially higher value in limiting community transmission of infectious diseases than doing without [16–19]. Also, facemask wearing was documented as one of the tools for curbing viral transmission from asymptomatic individuals to the population, thus limiting the epidemic’s growth rate or the reproductive number of the virus [19]. In order to limit community’s spread of COVID-19, community-wide use of facemasks was encouraged as a good and viable prevention and control measure [20,21]. This was because facemasks also served as visible sign of a widely prevalent respiratory pathogen, SARS-CoV-2, and one of the tools that reminded people of the importance of other infection control measures, such as social distancing [22]. Thus, facemasks were symbolic in that, beyond being tools, they increased healthcare workers’ perceived sense of safety, well-being, and trust in their healthcare systems [22].

A few African studies assessed the population’s perceptions and compliance with COVID-19 lockdown measures and the use of facemasks [23]. Some studies reported adequate COVID-19-related compliance among health workers, but others found significant gaps among the population [23].

As previously noted, handwashing with soap, water, sanitizers, and facemasks, limited human-to-human transmission of the virus when droplets were shed into the environment or on inanimate surfaces [8,9,24].

In a study to determine the adherence to all COVID-19 preventive measures in Uganda in the first wave of the SARS-CoV-2 outbreak, Bob OA *et al.* found that only 29% of participants adhered [25]. Yet, adherence to some measures was higher compared to others [25]. They reported that nearly all participants (96%) stated frequent handwashing with soap, but only 33% reported wearing a facemask in public [25].

Still, more sensitization regarding the importance of facemask wearing in containing the COVID-19 pandemic, subsidies, and free facemasks for those who were not able to afford were required.

Additionally, it was found that a population could stabilize the COVID-19 outbreak and halt the viral transmission by enforcing preventive measures, such as facemask wearing, hand hygiene, and physical distancing [25,26]. This finding was observed when the State of Arizona in the USA enforced the wearing of facemasks and other preventive measures; and cases of COVID-19 were reduced by 75% in a month [26].

**Fig 1:**
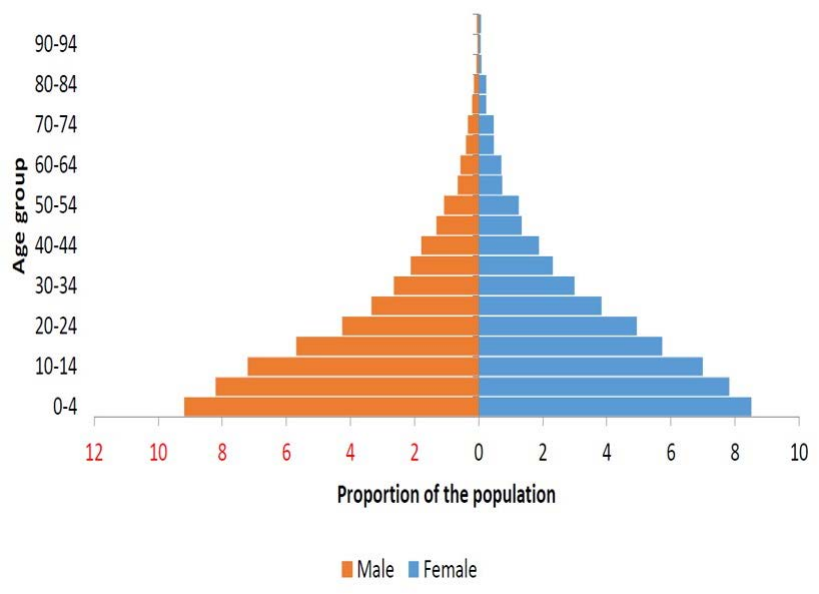
Age structure of Uganda’s population as of 2021, plotted by males and females.

In Uganda, there was a community transmission of COVID-19 which resulted in a geometrical spread of the virus across most districts in 2021 with resultant public health, economic, and socio-political implications [25,27]. The demographic characteristics and silhouettes of Ugandan population has a wide base with a very thin apex at about 86 years, meaning the majority of Ugandan population is young and less than 30 years (Fig 1).

**Fig 2:**
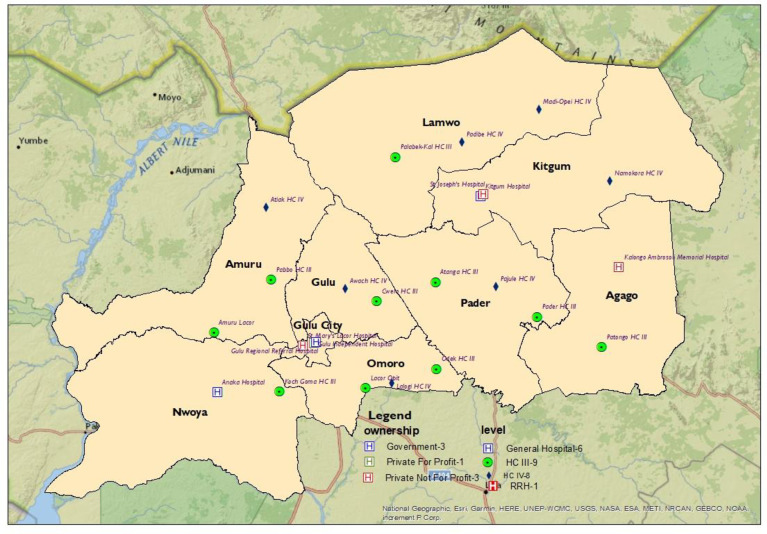
A map of the Acholi subregion, northern Uganda showing the study sites.

In northern Uganda, where this study was conducted (Fig 2), facemask wearing before the COVID-19 pandemic was majorly a preserve of health workers in clinical and public health sectors and very rare in the general population. Indeed, facemask wearing was not a common hygiene practice in the general population of Uganda. That being the case, it was necessary to determine the prevalence and correlates of facemask wearing among the general population of northern Uganda as a key for the roll-out of COVID-19 prevention measures with recommendations of the WHO, US. CDC and Ugandan Ministry of Health.

This study aimed to determine the prevalence and correlates of facemask wearing during the COVID-19 pandemic among adult populations of northern Uganda. **Source of Graph in Uganda Bureau of Statistics (UBOS)**

## Methods

### Ethics statement

The St. Mary’s Hospital, Lacor Institutional Review and Ethics Committee (LHIREC, No.0192/10/2021) approved this study. In addition, this study followed institutional guidelines where we obtained written informed consent from each respondent [28]. Furthermore, personal information was maintained confidential by not including personal identifiers on research documents. We kept all de-identified data under lock and key and residual data was archived in the Faculty of Medicine of Gulu University [28].

### Study design and context

This study was part of a larger research to determine compliance with the lockdown measures and other public health interventions during the COVID-19 pandemic in Uganda [28]. We conducted a cross-sectional study in northern Uganda between October and November 2021. The study was in nine districts of the Acholi subregion (Gulu, Gulu City, Amuru, Nwoya, Omoro, Pader, Agago, Kitgum, and Lamwo districts) [28,29]. The Acholi subregion has just emerged from a 20-year-old civil war between the Government of Uganda and the rebel, Lord’s Resistance Army (LRA) [30]. The total estimated population is two million three hundred thousand people in a total land surface area of 28,500km^2^ [29–32].

The study was conducted when the government of Uganda had eased the lockdown measures to COVID-19’s second wave. By then the number of COVID-19 patients had significantly reduced at treatment centres in northern Uganda [33]. Also, during the period, district taskforces set up by the Government of Uganda along administrative structures to tackle COVID-19 (national, districts, and communities) met weekly to discuss new developments and preventive action plans [33,34]. In addition, the President announced new work methods in public settings, whereby only 30% of staff in public and private organisations were allowed physically in offices [33,34]. These COVID-19 control measures were intended to disrupt day-to-day contact between management and communities to interrupt the cycle of physical person-to-person contact to break the transmission cycle of COVID-19 [33,34].

### Study sites

he study was conducted in the twenty-four health facilities in the Acholi subregion which were fairly distributed according to administrative structures in Uganda (Fig 2).

### Study respondents and sampling techniques

587 adult respondents were recruited from 24 selected high-volume health facilities using a systematic sampling method [28,35,36]. The twenty four health facilities were selected for ease of accessing participants, some of whom were coming to receive the free COVID-19 vaccines being offered to the general population [28]. In the outpatient departments (OPDs) of health facilities, every third participant was selected from the OPD registers until the target sample size was reached. Given the significant decline in the number of people accessing health facilities at the time, no fixed sample size was assigned to each facility. Instead, recruitment remained open until the overall study sample size was achieved. The study coordinator monitored daily questionnaire completions across the 24 sites via phone calls and concluded the study once the sample size was met.

### Sample size

A Raosoft sample size calculator was used for calculating our sample size. The computation was based on a 50% response distribution, 5% margin of error, and 95% Confidence Interval (CI). This online software is widely used for sample size estimation [37,38]. The assumption was that there was a total eligible population size of 50,000 (12.5% of the total adults above 18 years old in the Acholi subregion) in the nine districts of the Acholi subregion who attended the OPD services in one month. The minimum sample size grounded on these assumptions and factoring in a 10% non-response rate was 437 participants.

### Selection criteria

We included attendants and attendees of outpatients in the twenty-four health facilities in the Acholi subregion in the study period. Second, only respondents who were eighteen years and above were included. Respondents who did not consent or were unable to participate in all parts of the study and were not residents in the subregion six months before the study were excluded.

### Data collection

We conducted pretesting of the questionnaire in the outpatient department of Gulu Regional Referral Hospital (GRRH). The pretest results were not incorporated in the final data analysis. However, the questions achieved an internal validity of Cronbach’s α = 0.72. Thus, after obtaining written informed consent, an interviewer-guided questionnaire was presented to respondents in a face-to-face interview in the Outpatients’ department room, ensuring that infection, prevention, and control (IPCs) and standard operating procedures (SOPs) for COVID–19 were followed for respondents and interviewers [28,39]. At the time, the population was in fear and apprehension due to the distress of contracting COVID-19 and were not willing to receive researchers in their offices or homes [28]. Furthermore, we adopted a face-to-face questionnaire interview despite the risks of contracting COVID-19 because we wanted higher response rates [28]. We could have used an online approach for data collection however, previous surveys conducted in northern Uganda showed very few online and internet users (23%) [40] and mainly among ineligible participants for this study because of age. Online data collection would have also delayed study completion due to limited monitoring.

At each of the twenty-four health facilities, consented adult persons (≥18 years) who were OPD attendees and attendants were the target for recruitment to the study between 9:00 am and 6:00 pm daily, from Monday to Saturday until the sample size was achieved [28]. An interview which lasted between 30-40 minutes in a convenient room in the OPD was conducted by our research team [28]. Although the questionnaire was in English, only a few respondents required some questions to be translated into a local language (5/587, 0.85%).

Respondents who could not speak (due to speech disability but not language barrier) and those not residents in the region six months before the study were excluded. Data from the questionnaires were manually entered into excel by research assistants, verified for accuracy by the study coordinator, and later exported for statistical analysis.

### Data analysis

e analysed this data using Stata 18 [41] and used Microsoft Excel 2019 to generate graphs [28]. We conducted a descriptive analysis of respondents’ characteristics, presenting findings as proportions and percentages [28]. We assessed the prevalence of facemask wearing among respondents and presented findings as frequencies and bar charts. From the literature on COVID-19 facemask wearing during the pandemic, we selected independent variables for example, age, sex, occupation, level of education, employment status, race, nationality, tribes, religion, districts, addresses, comorbidity, smoking, drinking status, and marital status. The dependent variable was compliance with facemask wearing in public, which was defined as respondents reporting fidelity to always wearing facemasks in public. These responses were recorded as “yes” (1) or “no” (0).

We then applied a bivariate modified Poisson Regression analysis with robust confidence intervals to examine the relationship between each independent and dependent variable. Results were presented as crude prevalence ratios (cPR) and their respective P values at 95% Confidence Intervals (CI). We checked the collinearity between independent and dependent variables using the variance inflation factor (VIF) method, and all VIF values were below two (2), indicating no evidence of collinearity. Next, we fitted a multivariable Poisson regression model by entering all the selected independent variables to determine the factors associated with facemask wearing among the study population. The results were reported as adjusted Prevalence Ratios (aPR) with their respective P values and 95% Confidence Intervals. We considered a p-value ≤0.05 as statistically significant.

### Data credibility

To ascertain the credibility of our data, we undertook further analysis to explore the relationship between perceptions of facemask wearing (discomfort, inconvenience, attractiveness, and scepticism about COVID-19 protection) and self-reported facemask use in public settings by the study population. The findings demonstrated a clear link, with individuals reporting these concerns and were less likely to report regular facemask use. This, therefore, suggests some level of truthfulness in self-reported facemask use in our study population (S1 Table). Second, while we opted not to adjust for potential reporting bias (self-reported facemask wearing versus direct visual observation) using methods like Bonferroni correction, we revisited the data analysis based on findings in S2 Table. We applied the Bonferroni correction to the p-values to assess its impact on the statistical significance of each independent variable. Notably, all previously significant findings remained significant except for marital status (p = 0.05), which we included in the multivariable model based on a priori considerations (S2 Table).

## Results

We conducted a study on the adult population (≥18 years) in northern Uganda with a questionnaire response of 587/589(99.7%). Five respondents, 5/587(0.85%) required additional translation of the questionnaire from English to Acholi and two respondents declined to participate in the study 2/589(0.34%).

### Prevalence of facemask wearing

**Fig 3:**
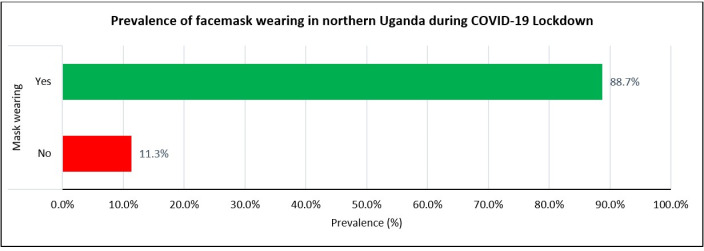
Prevalence of facemask-wearing during the COVID-19 lockdown in northern Uganda between October and November 2021.

This study presents a unique finding where a community that does not habitually wear facemasks faced the COVID-19 pandemic when there was a need to adjust behaviours and accept constraints to overcome the virus. Other than health workers in clinical and public health sectors, facemask wearing was rare among the general population of northern Uganda until the COVID-19 pandemic. However, because of the guidance from the Ugandan Ministry of Health and Presidential directives on facemask wearing in public settings, the population quickly accepted it and complied with the guidelines. The most substantial finding from this study was the self-reported high prevalence of facemask wearing in public settings among respondents [88.7%,95%CI:86%-91%] (Fig 3).

In Fig 3, 66(11.3%) reported not wearing facemasks, while 518(88.7%) reported always wearing facemasks as per national guidelines.

### Respondents’ characteristics

The study population was recruited from twenty-four health facilities in northern Uganda (Fig 2). Most participants were males, constituting more than half of the study population, 335(57.1%), and mainly in the age group of 25–34 years, 180(31.4%), who were either married or cohabiting, 341(58.9%). Also, most were Christians (94.9%), with Catholics being the predominant religion with over fifty percent, 312(53.2%). Regarding the tribes, the study area is predominantly Acholi ethnic group, 425(72.9%), and were from the districts of Gulu/Omoro, 220(37.5%). Although information from the Uganda Demographic Health Surveys (UDHS) [28,31] and Uganda Bureau of Statistics (UBOS) [29,31,32] showed that most population of the Acholi subregion had an adult literacy index of about 54%, most of our respondents had attained a tertiary level of education, 261(44.5%) (Table 1). They were employed mainly in the informal sector 149(25.4%). Even though there are several refugees, tourists, traders, and visitors in northern Uganda, most of our respondents were Ugandans, 581(99%) who did not use alcohol, 401(69.0%); did not smoke cigarettes, 545(94.1%); had no diabetes, 571(97.3%); had no heart diseases, 571(97.3%); were not obese, 578(98.5%); had no hypertension, 559(95.2%); had no Asthma, 572(97.4%); were not HIV positive, 577(98.3%); and had no other chronic diseases, 542(92.3%) (Table 1).

**Table 1:** Sociodemographic and health background characteristics of respondents from northern Uganda during the COVID-19 pandemic between October and November 2021.

Sociodemographic characteristics		n (%)
**Sex**	Female	252(42.9)
	Male	335(57.1)
**Age in years**	<25	150(26.2)
	25-34	180(31.4)
	35-44	157(27.4)
	≥45	86(15.0)
**Marital Status**	Married/cohabiting	341(58.9)
	Unmarried/others	238(41.1)
**Religion**	Catholic	312(53.2)
	Protestant	245(41.7)
	Others	30(5.1)
**Tribe**	Acholi	425(72.9)
	Lango	41(7.0)
	Others	117(20.1)
**Districts**	Gulu-Omoro	220(37.5)
	Kitgum-Lamwo	133(22.7)
	Amuru-Nwoya	92(15.7)
	Agago-Pader	86(14.7)
	Others	56(9.5)
**Level of formal education**	Tertiary	261(44.5)
	Secondary	225(38.3)
	Primary	64(10.9)
	None	37(6.3)
**Occupations**	Health professional	136(23.2)
	Agriculture/self-employed	115(19.6)
	Employed/retired	82(14.0)
	Student/unemployed	105(17.9)
	Others	149(25.4)
**Nationality**	Ugandan	581(99.0)
	non-Ugandan	6(1.0)
**Race**	African	586(99.8)
	Caucasian	1(0.2)
**Health background information**		
**Alcohol use**	No	401(69.0)
	Yes	180(31.0)
**Smoking status**	No	545(94.1)
	Yes	34(5.9)
**Diabetes Mellitus**	No	571(97.3)
	Yes	16(2.7)
**Heart diseases**	No	571(97.3)
	Yes	16(2.7)
**Obesity**	No	578(98.5)
	Yes	9(1.5)
**Hypertension**	No	559(95.2)
	Yes	28(4.8)
**Asthma**	No	572(97.4)
	Yes	15(2.6)
**HIV**	No	577(98.3)
	Yes	10(1.7)
**Other diseases**	No	542(92.3)
	Yes	45(7.7)
**Psychosocial and behavioral factors**		
Agreed with lockdown measures	No	80(13.6%)
	Yes	507(86.4%)
Desire to prevent infection spread	No	304(51.8%)
	Yes	283(48.2%)
Fear of contracting infection	No	183(31.2%)
	Yes	403(68.8%)
The fear of death	No	179(30.5%)
	Yes	408(69.5%)

### Facemask wearing at bivariate analysis

To study the relationship between the dependent (wearing facemasks) and independent variables (sociodemographic and health background characteristics), we conducted bivariate analyses between each independent variable and the dependent variable. We found that the following variables were statistically significant at 95% Confidence Intervals (CI) and p < 0.05; the unmarried respondents [(Crude Prevalence Ratio), cPR = 1.06,95%CI:1.00–1.12;p = 0.050]; obese [aPR = 1.10,95%CI:1.10–1.16;p < 0.010]; and respondents who agreed to the lockdown measures, [cPR = 1.22, 95%CI:1.07–1.39;p < 0.010] (Table 2).

**Table 2:** Bivariate analysis of respondents’ variables and facemask wearing in northern Uganda during the COVID-19 pandemic between October and November 2021.

Independent variables		Crude PR	95% CI	P value
Sociodemographic factors				
Sex	Female	Reference		
	Male	0.97	0.92-1.03	0.380
Age (years)	<25	Reference		
	25-34	0.96	0.89-1.04	0.300
	35-44	0.98	0.91-1.06	0.650
	≥45	0.97	0.89-1.07	0.590
Marital Status	Married/cohabiting	Reference		
	Unmarried/others	1.06	1.00-1.12	0.050^***^
Level of education	Tertiary	Reference		
	Secondary	1.02	0.96-1.09	0.450
	Primary	0.99	0.89-1.10	0.840
	None	0.92	0.78-1.08	0.290
Occupation	Health professionals	Reference		
	Agriculture/self-employed	1.02	0.93-1.11	0.710
	Student/unemployed	1.06	0.98-1.15	0.160
	Employed/retired	1.03	0.90-1.10	0.930
	Others	0.98	0.89-1.07	0.590
Alcohol consumption	No	Reference		
	Yes	0.98	0.92-1.05	0.640
Smoking	No	Reference		
	Yes	1	0.88-1.13	0.950
**Underlying health conditions**				
Diabetes	No	Reference		
	Yes	0.99	0.82-1.19	0.880
Heart disease	No	Reference		
	Yes	0.91	0.72-1.16	0.460
Obesity	No	Reference		
	Yes	1.10	1.10-1.16	<0.010^***^
Hypertension	No	Reference		
	Yes	0.84	0.68-1.04	0.110
Asthma	No	Reference		
	Yes	0.75	0.52-1.07	0.110
HIV	No	Reference		
	Yes	0.9	0.66-1.23	0.510
**Psychosocial and behavioral factors**				
Agreed with lockdown measures.	No	Reference		
	Yes	1.22	1.07-1.39	<0.010***
Desire to prevent infection spread.	No	Reference		
	Yes	1.03	0.97-1.09	0.320
Fear of contracting infection	No	Reference		
	Yes	1.01	0.96-1.07	0.650
The fear of death	No	Reference		
	Yes	1.02	0.96-1.09	0.460

***significant factors at 95%CI with p≤0.05.

### Correlates of facemask wearing in the study population

t the Multivariable Poisson Regression Analysis, we found that obese respondents were 1.12 times more likely to wear facemasks in public settings than those who were not, [adjusted Interval Rates Ratios, aPR = 1.12,95%CI:1.04–1.19;p < 0.01], and respondents who agreed to the lockdown measures during the pandemic were 1.23 times more likely to wear facemasks than those who did not, [aPR = 1.23,95%CI:1.07–1.41;p < 0.01]. The other sociodemographic and health background characteristics such as sex, age, occupation, level of education, religion, tribes, marital status, nationality, race, and comorbidities were not statistically significant at 95% Confidence Intervals (CI) (Table 3).

**Table 3:** Factors associated with facemask wearing in northern Uganda during the COVID-19 pandemic between October and November 2021.

Independent Variables		Crude PR	95% CI	P value	Adjusted PR	95% CI	P value
**Sex**	Female	**Reference**			**Reference**		
	Male	0.97	0.92-1.03	0.38	1.00	0.94-1.06	0.92
**Age category (years)**	<25	**Reference**			**Reference**		
	25-34	0.96	0.89-1.04	0.30	0.98	0.90-1.07	0.71
	35-44	0.98	0.91-1.06	0.65	1.03	0.95-1.11	0.54
	≥45	0.97	0.89-1.07	0.59	1.02	0.92-1.12	0.73
**Marital Status**	Married/cohabiting	**Reference**			**Reference**		
	Unmarried/others	1.06	1.00-1.12	0.05	1.06	0.99-1.13	0.10
**Obesity**	No	**Reference**			**Reference**		
	Yes	1.1	1.10-1.16	<0.01	1.12	1.04-1.19	<0.01^***^
**Agreed with lockdown measures**	No	**Reference**			**Reference**		
	Yes	1.22	1.07-1.39	<0.01	1.23	1.07-1.41	<0.01^***^

*Age and sex were taken as priory in the multivariable analysis, ***significant factors at 95%CI with p≤0.05.

In Table 1, most respondents were males, 335(57.1%); age-group of 25–34 years, 180(31.4%); married/cohabiting, 341(58.9%); Catholics, 312(53.2%); Acholi 425(72.9); from Gulu/Omoro districts, 220(37.5); with tertiary level of education, 261(44.5%); other occupations, 149(25.4%); Ugandans, 581(99%); did not use alcohol, 401(69.0%); did not smoke cigarettes, 545(94.1%); had no diabetes, 571(97.3%); had no heart diseases, 571(97.3%); were not obese, 578(98.5%); had no hypertension, 559(95.2%); had no Asthma, 572(97.4%); were not HIV positive, 577(98.3%); and no other diseases, 542(92.3%).

Table 2 shows variables that were significantly associated with facemask wearing at bivariate analysis; the unmarried with [Crude Prevalence Ratio, cPR = 1.06, 95%CI:1.00–1.12; p = 0.050]; Obese respondents, [cPR = 1.10,95%CI:1.10–1.16;p < 0.010]; and respondents who agreed to the lockdown measures, [cPR = 1.22, 95%CI:1.07–1.39;p < 0.010].

Table 3 shows factors significantly associated with facemask-wearing during the COVID-19 pandemic in northern Uganda. These were; obese respondents, [adjusted Prevalence Ratios, aPR = 1.12,95%CI:1.04–1.19;p < 0.01], and respondents who agreed with the lockdown measures, [aPR = 1.23, 95%CI:1.07–1.41;p < 0.01].

## Discussion

Facemask wearing in public settings where social distancing measures were problematic to maintain has been documented as one of the most important prevention measures that limited the acquisition and spread of COVID-19 by the WHO and the US CDC [11,12]. Thus, the WHO and US CDC developed guidelines for facemasks use in public environments during the COVID–19 pandemic [11,12]. In addition, epidemiological evidence showed that facemask wearing was effective in preventing the transmission of COVID-19 [42–45]. Our study population showing an age structure of Ugandans as of 2021 plotted for males and females (Fig 1) and the Acholi subregion of Northern Uganda (Fig 2) presents an interesting finding.

Our study found an optimum prevalence of community facemask wearing during the COVID-19 pandemic (88.7%) in northern Uganda (Fig 3). The study showed that facemask wearing among adult community members in the Acholi subregion (Fig 2) was within acceptable range (Fig 3). This was in the backdrop of a postwar setting and a higher prevalence of poverty in northern Uganda (using UBOS, 2020 data) compared to the rest of Uganda [29–31]. Serial reports from the Uganda Bureau of Statistics (UBOS) [29–32] showed that northern Uganda had consistently posted worse poverty indicators (68%) than the rest of the country, and facemasks acquisition required money to purchase. Nevertheless, most Ugandan population needed help to acquire facemasks during the pandewere distributed free to a section of the population by the Government of Uganda (UBOS, 2020) [29].

Notably, the level of facemask wearing in our study population was inconsistent with another study in Uganda by Mboowa *et al*. [46]. In their study, they reported a prevalence of facemask wearing of 70.3%, which was lower than ours at 88.7% (Fig 3). However, our level of facemask wearing was far higher than that of Amodan and colleagues at (33%) [47] and lower than Ssebuufu *et al.* (99.3%) [48]. These differences could have been because Amodan and colleagues [47] conducted their study in Uganda at the earlier stage of the pandemic before initiatives such as mass distribution of facemasks, sensitization, mobilization, engagement of communities [49], and the strict enforcement of facemask use in public places by security forces were introduced [49–53]. The prevalence of facemask wearing in Ssebuufu *et al.* [48] was higher than ours, partly because it did not use face-to-face interviews or guilt-free questions when assessing participants’ self-reported practices as we did [53]. Thus, findings on facemask-wearing in Uganda may reflect the participants and the period of the pandemic when the study was conducted. In addition, Ssebuufu *et al’s,* participants were Ugandan community members who were recruited by an online snowballing techniques [48], thus presenting potential risks of selection biases and non-probability sampling of respondents. Thus, the prevalence of facemask-wearing could vary according to the study participants, knowledge levels, attitudes, practices, exposures, and risk perceptions. Therefore, this acceptable level of community facemask wearing among adult community members in northern Uganda (Table 1) emphasizes the need for initiatives to scale up the practice now and in the future to prevent and control any emerging infectious respiratory diseases (Tables 2 and 3).

We acknowledge that our study lacked a comparison between self-reported and direct visual observation of the population on facemask wearing in public settings during the period. Studies have shown that there is a huge disparity between self-reported facemask wearing compared to direct visual observation [54,55]. Self-reported facemask wearing tends to be higher than the visually observed [54,55]. Self-reported facemask wearing has shortcomings of over-reporting and over-representation as observed in some developing countries [54,55]. However, we note that at the time of this study, facemask wearing was mandatory in all health facilities in Uganda and all study participants we interviewed wore facemasks as per the IPC and SOPs guidelines in Uganda [28].

From a global health perspective, our finding shows that the prevalence of facemask wearing in public in northern Uganda was comparable to many communities in Asian countries where the practice is part of their culture. For example, our study found that facemask wearing during the COVID-19 pandemic in northern Uganda was much higher than in Hong Kong [56], Malaysia [57], and Ghana [58] at 69.2%, 51.2%, and 32.3%, respectively. Ours was high (above 80%) like other studies in Uganda [59,60] and Pakistan [61] where they found that 99.3%, 93%, and 93.9% of respondents, reported wearing facemasks during the COVID-19 pandemic when in public, respectively.

Many factors may explain these findings. First, the belief and practice that facemasks could protect them against COVID-19. Second, the strict enforcement of facemask wearing in Uganda by security forces gave no room for defaulting [50–52]. Third, the government of Uganda supplied facemasks free to community members across the country, so access to facemasks was not a problem. Fourth, because the kind and quality of facemasks did not matter in Uganda, some community members used clothes or handkerchiefs to cover their faces while in public, and these were accepted as facemasks. Also, our study assessed compliance and satisfaction with facemask wearing as an appropriate COVID-19 prevention measure after Uganda’s second and most severe wave of COVID-19 outbreak. 88.7% of our study population reported wearing facemasks in public settings during the pandemic (Fig 3). In addition, the level of satisfaction with facemask use was higher at (90%).

This is an important information for northern Uganda as it was previously estimated that proper facemask wearing with a coverage of 80% would halt the transmission of the virus [26]. This was confirmed when the State of Arizona in the USA enforced facemask-wearing and other preventive measures; COVID-19 cases were reduced by 75% in a month [26].

Like other countries in the African continent, facemask wearing in Uganda is not a common hygiene practice but it was introduced to the African communities in response to the COVID-19 pandemic [61,62]. In addition, a lower facemask use in some communities in the African continent could have resulted from initial information needing to be more consistent about the importance of facemask wearing for preventing COVID-19 transmission to the general population [61,62]. In addition, there was information circulating in many communities that the threat posed by COVID-19 to Ugandans and African populations was likely milder considering a warmer tropical environment, less crowded environment, and a predominantly younger population structure [10,63]. The quick adoption of facemask wearing in public settings among the study population during the pandemic was commendable and worth reporting.

In contrast to our findings in northern Uganda, many communities in the African continent did not wear facemasks during the pandemic because they were uncomfortable or because they did not think they were necessary [64]. Further, our study found that correlates of facemasks wearing were respondents with comorbidity (obesity) and those who agreed with the lockdown measures instituted by the government of Uganda to curb the exponential spread of COVID-19 (Tables 1–3). This finding is consistent with many studies that show that a population with high-risk perceptions were more likely to adhere to facemask wearing as a preventive measure for the control of infectious diseases such as COVID-19 [33,34,43,46,47]. In addition, the comorbid population was a special target group by the Ugandan Ministry of Health campaigns during the pandemic with specific emphasis on preventing these high-at-risk population from acquiring the virus, developing severe disease, hospitalization, and death [33,34,43,46,47]. Furthermore, the Ugandan Ministry of Health prioritized the high-at-risk community members with supplies of facemasks, vaccines, and awareness campaigns on the dangers posed by COVID-19 [33,34,43,46,47].

The high-risk perceptions among obese respondents in northern Uganda were rooted in a higher COVID-19 mortality observed among obese patients seen in one of the health facilities in Uganda than those who were not [64]. A mortality risk study conducted in Wuhan, China showed that comorbidity could impact the share of mild cases that developed severe symptoms [65]. In Asia and Europe for example, hypertension, obesity, diabetes, and coronary heart diseases have been drivers of adverse health outcomes from COVID-19 [65,66]. The combined prevalence of diabetes, hypertension, and obesity was higher in regions that were used to derive the recovery rates when baseline simulations had already been accounted for, compared to Ghana, Kenya, and Senegal where other comorbidities afflict them. For example, Ghana, Kenya, and Senegal have persistent and higher rates of anemia, and Tuberculosis (TB) [67]. However, there were no studies on the magnitude and impact of anemia, TB, HIV, and AIDs on the recovery of patients with COVID-19. Thus, there are more considerations to be made as more information about the adverse consequences of comorbidities in different settings are being considered. Because of the many uncertainties about COVID-19, participants with comorbidities needed to consider protecting themselves by consistently wearing facemasks. We, the authors propose further studies on the trending of comorbidity in COVID-19 pathogenesis to reach a definite conclusion.

Our findings that facemask wearing was more likely among respondents who agreed with the lockdown measures were consistent with many studies in Uganda [68–71]. The lockdown measures for COVID-19 prevention and control were implemented in Uganda during the first and second waves of the pandemic [68–71]. In both waves, lockdown measures were instituted through presidential directives strictly enforced by Uganda’s security forces [50,51,68–70]. So, facemask wearing was mandatory when an individual was in public settings, and that could explain the high coverage in our study findings [50,51]. In addition, northern Uganda is in a postwar era where the experience of war and the use of security forces to enforce government directives were fresh in the minds of the population [30]. It is likely that some community members wore facemasks not because of conviction or belief that it was helpful but rather the fear that one would be apprehended or punished by the security forces [33].

Inconsistent with our study findings, another in Somalia found that only half of their respondents reported wearing facemasks during the COVID-19 pandemic [10]. This was far below the 80% threshold recommended by modeling studies in England and Whales [72], and thus the need for facemask wearing to be improved in Somalia [10]. Of particular note, reusable cloth facemasks were reported only in about one-quarter of the participants in Somalia [10]. Cloth facemasks, however, offer some advantages, such as lower costs and being far less dangerous for the environment when compared to surgical masks, and have been proposed for COVID-19 control, especially in resource-poor settings [71–73]. Aside from the reported difficulties of acquiring masks (such as lack of finances and not knowing where to obtain masks), as also observed in our study, other significant limitations to wearing facemasks included the associated discomfort and the widespread thoughts that masks were unnecessary in times of COVID-19. Indeed, facemask-wearing is a relatively odd practice among the African population, and they should be educated about its importance during the COVID-19 crisis. This finding contrasts with Asian countries, where facemasks are culturally accepted as a common hygiene practice [74].

Findings from this study could help inform policy on facemask wearing in communities where masks are culturally not part of their hygiene practices, however since participants were recruited from health facilities, the findings may not be generalizable to the broader population. The cross-sectional nature of this study also comes with inherent limitations of not measuring variables over time, thus the risk of not capturing the dynamism of changing times and perceptions of respondents (S1 Table); (S2 Table). Likewise, we only captured the views and opinions of adult respondents ≥ 18 years old and not below 18 years old. Additionally, this study did not directly observe respondents on facemask wearing in other public settings except in the OPD, where facemask wearing was mandatory. The lack of visual observation could introduce bias of over-estimation and over-reporting of self-reported wearing of facemasks as has been observed in studies on the African continent [54].

Finally, our findings that most respondents in the study population had attained a tertiary level of education pose a challenge of representation (Table 1). Information obtained from studies in northern Uganda shows that most of the population does not have tertiary education [25,34]. This finding may present a selection bias in our study population. However, these findings may be generalized to rural communities in sub-Saharan Africa with similar contexts.

## Conclusions

The most significant finding of this study was the high prevalence of self-reported facemask wearing among adult community members in northern Uganda. The correlates of facemask wearing were obese respondents and those who agreed with the presidential directives on lockdown measures. Although this prevalence is within acceptable rates, the strict enforcement of the practice by security forces has raised concerns among many community members and human rights advocates. We recommend more studies on communities’ perspectives on the challenges and benefits of facemask-wearing after the COVID-19 pandemic.

## Supporting information

S1 Table Responses of participants on adherence to facemask wearing in public. S1 Table shows there was truthfulness in self-reported facemask use in our study population.
(DOCX)

S2 Table The Bonferroni corrected P-values for independent variables. S2 Table shows the Bonferroni correction to the p-values and its impact on the statistical significance of each independent variable. All previously significant findings remained significant except for marital status.
(DOCX)

## Abbreviations:

AIDS: Acquired immune deficiency syndrome
HIV: Human immunodeficiency virus
HC: Health centre
CI: Confidence intervals
COVID-19: Coronavirus disease-19
LHIREC: Lacor Hospital institutional review and ethics committee
IPC: Infection, prevention, and control
OPD: Outpatient department
SOPs: Standard operating procedures
TB: Tuberculosis
UBOS: Uganda bureau of statistics
UDHS: Uganda demographic health surveys
US CDC: United states of america centers for disease control and prevention
WHO: World health organization
