# Supplementary material for: Prevalence and correlates of facemask usage during the second wave of COVID-19 pandemic in Uganda
Source: PLOS Glob Public Health. 2025 Feb 7;5(2):e0002569. doi: 10.1371/journal.pgph.0002569 (PMC11805370; doi:10.1371/journal.pgph.0002569)
Supplement: S1 Table — shows there was truthfulness in self-reported facemask use in our study population. (DOCX) [file pgph.0002569.s001.docx]

**S1 Table: Participants' responses on adherence to facemask wearing in public in northern Uganda between October and November of 2021.**

| Adhered to facemask wearing in public | **Perceptions of Facemask Wearing in Public** | | | | | | |
| --- | --- | --- | --- | --- | --- | --- | --- |
|  | Masks are inconvenience | Cases | Controls | OR | 95% conf. | | P value |
|  | No | 275 | 42 | 6.55 | 4.7 | 9.1 | 0.106 |
|  | Yes | 243 | 24 | 10.13 | 6.7 | 15.4 |  |
|  | Masks suffocate | Cases | Controls | Odds |  |  |  |
|  | No | 181 | 33 | 5.5 | 3.8 | 7.9 | 0.017 |
|  | Yes | 337 | 33 | 10.2 | 7.1 | 14.6 |  |
|  | Masks take away my beauty | Cases | Controls | Odds |  |  |  |
|  | No | 442 | 50 | 8.84 | 6.6 | 11.8 | 0.045 |
|  | Yes | 76 | 16 | 4.75 | 2.8 | 8.1 |  |
|  | Masks do not help | Cases | Controls | Odds |  |  |  |
|  | No | 497 | 57 | 8.71 | 6.6 | 11.5 | 0.001 |
|  | Yes | 21 | 9 | 2.33 | 1.1 | 5.1 |  |
