# Supplementary material for: Prevalence and correlates of facemask usage during the second wave of COVID-19 pandemic in Uganda
Source: PLOS Glob Public Health. 2025 Feb 7;5(2):e0002569. doi: 10.1371/journal.pgph.0002569 (PMC11805370; doi:10.1371/journal.pgph.0002569)
Supplement: S2 Table — shows the Bonferroni correction to the p-values and its impact on the statistical significance of each independent variable. All previously significant findings remained significant except for marital status. (DOCX) [file pgph.0002569.s002.docx]

**S2 Table: The Bonferroni corrected P-values for independent variables on Facemask wearing in public in Northern Uganda between October and November 2021.**

| **Independent variables** | Crude IRR | 95% CI | P value | Bonferroni corrected P value | Significant (Bonferroni) |
| --- | --- | --- | --- | --- | --- |
| **Sociodemographic factors** |  |  |  |  |  |
| Sex |  |  |  |  |  |
| Female | Reference |  |  |  |  |
| Male | 0.97 | 0.92-1.03 | 0.380 | 0.380 | No |
| **Age (years)** | | | | | |
| <25 | Reference |  |  |  |  |
| 25-34 | 0.96 | 0.89-1.04 | 0.300 | 0.300 | No |
| 35-44 | 0.98 | 0.91-1.06 | 0.650 | 0.650 | No |
| ≥45 | 0.97 | 0.89-1.07 | 0.590 | 0.590 | No |
| **Marital Status** | | | | | |
| Married/cohabiting | Reference |  |  |  |  |
| Unmarried/others | 1.06 | 1.00-1.12 | 0.050 | 0.050 | No |
| **Level of education** | | | | | |
| Tertiary | Reference |  |  |  |  |
| Secondary | 1.02 | 0.96-1.09 | 0.450 | 0.450 | No |
| Primary | 0.99 | 0.89-1.10 | 0.840 | 0.840 | No |
| None | 0.92 | 0.78-1.08 | 0.290 | 0.290 | No |
| **Occupation** | | | | | |
| Health professionals | Reference |  |  |  |  |
| Agriculture/self-employed | 1.02 | 0.93-1.11 | 0.710 | 0.710 | No |
| Student/unemployed | 1.06 | 0.98-1.15 | 0.160 | 0.160 | No |
| Employed/retired | 1.01 | 0.90-1.10 | 0.930 | 0.930 | No |
| Others | 0.98 | 0.89-1.07 | 0.590 | 0.590 | No |
| **Alcohol consumption** | | | | | |
| No | Reference |  |  |  |  |
| Yes | 0.98 | 0.92-1.05 | 0.640 | 0.640 | No |
| **Smoking** | | | | | |
| No | Reference |  |  |  |  |
| Yes | 1.01 | 0.88-1.13 | 0.950 | 0.950 | No |
| **Underlying health conditions** | | | | | |
| **Diabetes** | | | | | |
| No | Reference |  |  |  |  |
| Yes | 0.99 | 0.82-1.19 | 0.880 | 0.880 | No |
| **Heart disease** | | | | | |
| No | Reference |  |  |  |  |
| Yes | 0.91 | 0.72-1.16 | 0.460 | 0.460 | No |
| **Obesity** | | | | | |
| No | Reference |  |  |  |  |
| Yes | 1.10 | 1.10-1.16 | <0.010 | <0.010 | Yes |
| **Hypertension** | | | | | |
| No | Reference |  |  |  |  |
| Yes | 0.84 | 0.68-1.04 | 0.110 | 0.110 | No |
| **Asthma** | | | | | |
| No | Reference |  |  |  |  |
| Yes | 0.75 | 0.52-1.07 | 0.110 | 0.110 | No |
| **HIV** | | | | | |
| No | Reference |  |  |  |  |
| Yes | 0.90 | 0.66-1.23 | 0.510 | 0.510 | No |
| **Psychosocial and behavioral factors** | | | | | |
| Agreed with lockdown measures | | | | | |
| No | Reference |  |  |  |  |
| Yes | 1.22 | 1.07-1.39 | <0.010 | <0.010 | Yes |
| Desire to prevent infection spread | | | | | |
| Yes | Reference |  |  |  |  |
| No | 1.03 | 0.97-1.09 | 0.320 | 0.320 | No |
| Fear of contracting infection | | | | | |
| Yes | Reference |  |  |  |  |
| No | 1.01 | 0.96-1.07 | 0.650 | 0.650 | No |
| The fear of death | | | | | |
| Yes | Reference |  |  |  |  |
| No | 1.02 | 0.96-1.09 | 0.460 | 0.460 | No |
